# Antimicrobial Photoinactivation Using Visible Light Plus Water-Filtered Infrared-A (VIS + wIRA) and *Hypericum Perforatum* Modifies *In Situ* Oral Biofilms

**DOI:** 10.1038/s41598-019-56925-7

**Published:** 2019-12-30

**Authors:** Andreas Vollmer, Ali Al-Ahmad, Aikaterini Argyropoulou, Thomas Thurnheer, Elmar Hellwig, Thomas Attin, Kirstin Vach, Annette Wittmer, Kerry Ferguson, Alexios Leandros Skaltsounis, Lamprini Karygianni

**Affiliations:** 1 Department of Operative Dentistry and Periodontology, Center for Dental Medicine, Albert-Ludwigs-University, Freiburg, Germany; 2 Department of Pharmacognosy and Chemistry of Natural Products, Faculty of Pharmacy, National and Kapodistrian University of Athens, Athens, Greece; 3 Clinic for Conservative and Preventive Dentistry, Center of Dental Medicine, University of Zurich, Zurich, Switzerland; 4 Institute for Medical Biometry and Statistics, Center for Medical Biometry and Medical Informatics, Albert-Ludwigs-University, Freiburg, Germany; 5 Institute of Medical Microbiology and Hygiene, Albert-Ludwigs-University, Freiburg, Germany; 6 Botanical Innovation, Unit 2, 390 Clergate Road, Orange, NSW 2800 Australia

**Keywords:** Biofilms, Dental diseases

## Abstract

Due to increasing antibiotic resistance, the application of antimicrobial photodynamic therapy (aPDT) is gaining increasing popularity in dentistry. The aim of this study was to investigate the antimicrobial effects of aPDT using visible light (VIS) and water-filtered infrared-A (wIRA) in combination with a *Hypericum perforatum* extract on *in situ* oral biofilms. The chemical composition of *H. perforatum* extract was analyzed using ultra-high-performance liquid chromatography coupled with high resolution mass spectrometry (UPLC-HRMS). To obtain initial and mature oral biofilms *in situ*, intraoral devices with fixed bovine enamel slabs (BES) were carried by six healthy volunteers for two hours and three days, respectively. The *ex situ* exposure of biofilms to VIS + wIRA (200 mWcm^−2^) and *H. perforatum* (32 mg ml^−1^, non-rinsed or rinsed prior to aPDT after 2-min preincubation) lasted for five minutes. Biofilm treatment with 0.2% chlorhexidine gluconate solution (CHX) served as a positive control, while untreated biofilms served as a negative control. The colony-forming units (CFU) of the aPDT-treated biofilms were quantified, and the surviving microorganisms were identified using MALDI-TOF biochemical tests as well as 16 S rDNA-sequencing. We could show that the *H. perforatum* extract had significant photoactivation potential at a concentration of 32 mg ml^−1^. When aPDT was carried out in the presence of *H. perforatum*, all biofilms (100%) were completely eradicated (p = 0.0001). When *H. perforatum* was rinsed off prior to aPDT, more than 92% of the initial viable bacterial count and 13% of the mature oral biofilm were killed. Overall, the microbial composition in initial and mature biofilms was substantially altered after aPDT, inducing a shift in the synthesis of the microbial community. In conclusion, *H. perforatum*-mediated aPDT using VIS + wIRA interferes with oral biofilms, resulting in their elimination or the substantial alteration of microbial diversity and richness. The present results support the evaluation of *H. perforatum*-mediated aPDT for the adjunctive treatment of biofilm-associated oral diseases.

## Introduction

The growing ineffectiveness of several conventional antimicrobial agents for the treatment of various biofilm-associated infections has been identified^1,2^. Interestingly, it has been shown that biofilm inhabitants have an enhanced resistance of up to 1,000 times against antibiotics compared with their planktonic counterparts^3,4^. Recently, aPDT was successfully applied to treat biofilm-associated oral infections such as caries, periodontitis and periimplantitis^5^. Thus, aPDT-driven oral decontamination can serve as an adjunct method to traditional dental treatment approaches.

The majority of aPDT protocols involve the combined application of various harmless light sources and mostly cationic local photosensitizing agents such as methylene blue (MB), toluidine blue O (TB), indocyanine green (ICG) and chlorine e6 (Ce6)^6–8^. Regarding the light sources, the combination of visible light (VIS) and water-filtered infrared-A (wIRA) has been shown to increase *in situ* temperature and perfusion levels, inducing wound healing at higher oxygen partial tissue pressure. Interestingly, VIS + wIRA correlates with significant pain reduction^9^. Another advantage of wIRA is the induction of low thermal stress via sufficient tissue penetration, which protects the external tissue layers^10,11^. Already-tested photosensitizers such as TB and Ce6 have shown enhanced antimicrobial activity in combination with aPDT against monospecies and multispecies biofilms^12–15^. Recently, VIS + wIRA in combination with TB or Ce6 has proven to be a potent alternative treatment against *in situ* supragingival and subgingival multispecies biofilms^16,17^.

The photodynamic properties of *Hypericum perforatum* (St. John’s Wort) have been well known for years now^18,19^. Indeed, it is one of the most commonly-investigated medicinal plants of the last two decades^20–22^. To date, *H. perforatum* has been widely used for topical application to skin prior to aPDT to accelerate chronic wound healing^18^. *H. perforatum* can also be used as an antidepressive substance^23,24^. Due to its additional favorable anti-oxidant, anti-inflammatory, anti-cancer and antimicrobial properties, *H. perforatum* is responsible for the enhanced differentiation of keratinocytes^18^. Interestingly, its high biocompatibility could lead it to play an outstanding role in aPDT application in the dental field^25^. Among *H. perforatum*’s main components, lipophilic hyperforin and hypericin seem to be the most active substances^26,27^. Favorably, *H. perforatum* shows no mutagenic potential and binding to DNA molecules^28^.

Despite their excellent properties, common photosensitizers such as TB or Ce6 fail to efficiently penetrate into extracellular DNA within the polysaccharide matrix to eradicate the pathogens situated in the deepest biofilm layers^29^. In this context, the photodynamic properties of *H. perforatum* prompted the idea that aPDT using VIS + wIRA and *H. perforatum* could be a promising alternative method for treating multispecies oral biofilms. Considering that the development of oral diseases such as periodontitis and periimplantitis relates to pathological shifts in oral biofilms, modifying biofilm composition and thus diversity could positively affect the treatment impact^23^.

**Figure 1:**
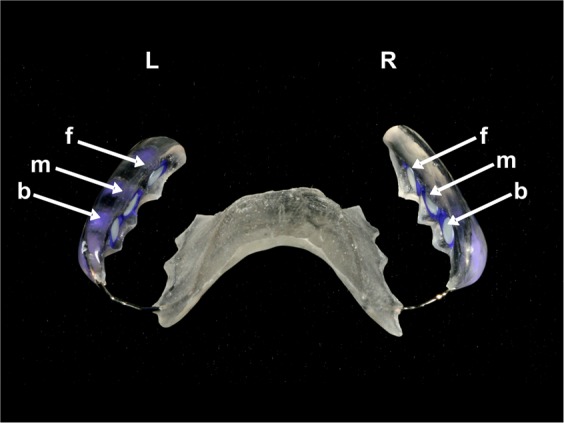
Individual upper jaw acrylic splint. The BES were attached at the front (f), in the middle (m) and at the back (b) on the right (R) and left (L) sides of the splint. Both BES downward-facing surface margins were covered with silicon, while only their upward-facing surfaces remained exposed to the buccal tooth surfaces during the experiments.

**Figure 2:**
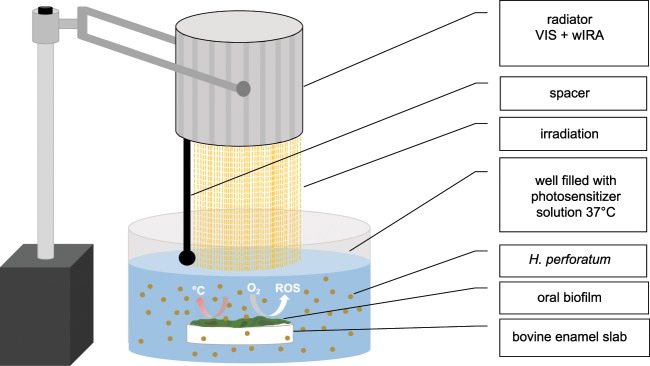
Graphical scheme depicting aPDT using VIS + wIRA as a light source and *H. perforatum* as a photosensitizer against oral biofilms. The VIS + wIRA with a broadband water-filtered spectrum (570–1400 nm) facilitated the excitation of the photosensitizing agent (*H. perforatum*), triggering its interaction with oxygen (O_2_). ROS were then released, eradicating both planktonic and adherent oral microorganisms.

The aim of the present study was to investigate the survival and diversity of microorganisms within initial and mature oral biofilms after the application of aPDT using VIS + wIRA and *H. perforatum* as a photosensitizer. For this approach, we cultivated oral biofilms *in situ* onto bovine enamel samples (Fig. 1) for two hours (h) and three days (d), respectively. The *in situ* biofilms were then treated with the photosensitizer *H. perforatum* and VIS + wIRA *ex vivo* aPDT (Fig. 2). To our knowledge, alteration in biofilm diversity following aPDT by VIS + wIRA and *H. perforatum* has not been studied to date. The null hypothesis was that aPDT using VIS + wIRA and *H. perforatum* would not eliminate oral biofilms or modify the composition of *in situ* oral biofilms.

## Results

### Chemical composition of the *H. perforatum* extract

**Figure 3:**
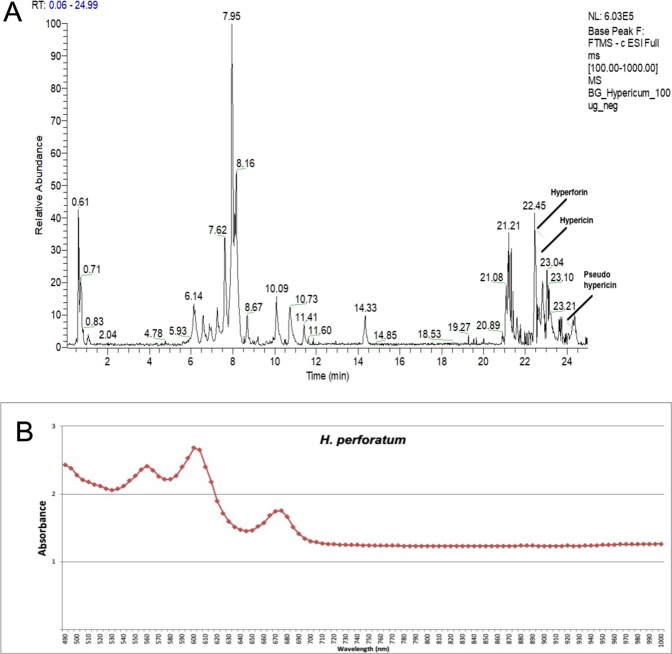
Panel A depicts UPLC-ESI-HRMS chromatogram of the *H. perforatum* extract. The peaks of hyperforin, hypericin and pseudohypericin are marked on the graph. Panel B depicts the absorbance spectrum of the fabricated *H. perforatum* extract including 16% alcohol. The concentration of *H. perforatum* was 32 mg ml^−1^. The measurement of the spectrum was conducted using a Tecan Infinite 200 reader (Tecan, Crailsheim, Germany).

A rapid and accurate method was developed, aiming to detect the major active compounds in the *H. perforatum* extract, as described elsewhere^30^. For the LC-HRMS, an Orbitrap high resolution mass analyzer was used in negative ionization mode. Peaks were identified by comparing the retention times and HRMS data of the compounds with already-published data^31,32^ and natural product databases (PubChem, ChemSpider). The extract of *H. perforatum* contains a wide range of constituents (Table 1, Fig. 3). All of the detected compounds along with their names, Rt (min), molecular formula, theoretical and experimental m/z, mass difference (ppm) and RDBeq (Relative Double Bond equivalent) value are summarized in Table 1, whereby a total of sixteen secondary compounds were detected. Phenolic acids, flavonoids, biflavones, phloroglucinols and naphthodianthrones were identified. Naphthodianthrones are characteristic compounds of the genus *Hypericum*. In the studied extract, hypericin and pseudohypericin were detected at Rt 22.74 and 23.27 min, respectively. Phloroglucinol – represented by hyperfirin and hyperforin – was also identified, as they are considered typical representatives of the genus. Several types of flavonoids – a flavonol (quercetin), flavonoid-*O*-glycosides (isoquercetrin, quercitrin, rutin and skyrin-2-*O*-glucopyranoside) and flavonol galactosides (hyperoside and isoquercitrin) – were identified. Two dimeric types of flavones – I3,II8 Biapigenin at Rt 11.41 min and amentoflavone at Rt 12.04 min – that are commonly detected in *H. perforatum* were also found in the studied extract. Additionally, various phenolic compounds (quinic acid, chlorogenic acid and 3-*O*-feruloylquinic acid) were detected.

**Table 1:** Chromatographic and spectrometric characteristics of secondary metabolites identified in *H. perforatum* by LC(ESI-)HRMS.

No	Rt (min)	EC [M-H]^−^	Experimental	Theoretical	Δm ppm	RDBeq	Identification of compound
			[M-H]^−^ *m/z*				
1	0.65	C_7_H_11_O_6_	191.0544	191.0561	−8.730	2.5	**Quinic acid**
2	6.14	C_16_H_17_O_9_	353.0835	353.0819	−12.363	8.5	**Chlorogenic acid**
3	7.60	C_17_H_19_O_9_	367.0990	367.0976	−12.137	8.5	**3-O-Feruloylquinic acid**
4	8.08	C_21_H_19_O_12_	463.0826	463.0823	0.615	12.5	**Hyperoside**
5	8.16	C_21_H_19_O_12_	463.0828	463.0823	1.077	12.5	**Isoquercitrin**
6	8.67	C_21_H_19_O_11_	447.0880	447.0874	−12.536	12.5	**Isoquercetrin**
7	8.72	C_21_H_19_O_11_	447.0877	447.0874	−12.334	12.5	**Quercitrin**
8	10.09	C_15_H_9_O_7_	301.0319	301.0295	−11.480	11.5	**Quercetin**
9	10.36	C_27_H_29_O_16_	609.1388	609.1402	−1.965	13.5	**Rutin**
10	11.41	C_30_H_17_O_10_	537.0762	537.0827	−12.195	22.5	**I3,II8 Biapigenin**
11	12.04	C_30_H_17_O_10_	537.0757	537.0827	−12.994	22.5	**Amentoflavone**
12	14.34	C_36_H_27_O_15_	699.1268	699.1355	−12.463	23.5	**Skyrin-2-*O*-glucopyranoside**
13	20.37	C_30_H_43_O_4_	467.3113	467.3167	−11.564	9.5	**Hyperfirin**
14	22.20	C_35_H_51_O_4_	535.3730	535.3793	−11.649	10.5	**Hyperforin**
15	22.74	C_30_H_15_O_8_	503.0715	503.0714	−11.352	23.5	**Hypericin**
16	23.27	C_30_H_15_O_9_	519.0661	519.0663	−11.723	23.5	**Pseudohypericin**

### aPDT effects on the viable counts of oral microorganisms at initial adhesion and in mature oral biofilms

**Figure 4:**
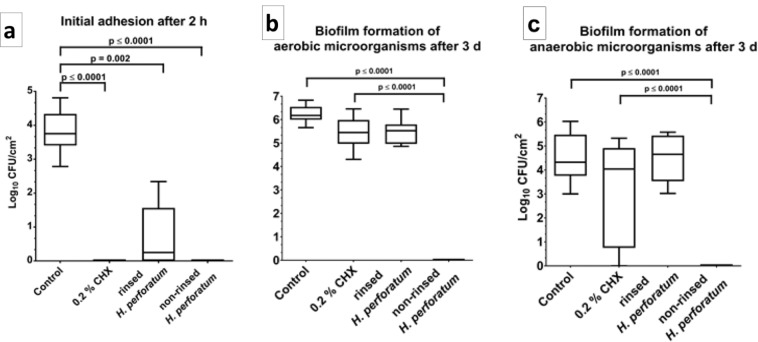
Boxplots of CFU counts, demonstrating the photodynamic efficiency (aPDT using VIS + wIRA and *H. perforatum*) against oral microorganisms after two-hour (2 h) initial adhesion (**a**) as well as against aerobic (**b**) and anaerobic (**c**) microorganisms following three-day (3 d) biofilm formation. The bacterial counts of the following treatment groups are demonstrated for two-hour- and three-day-old oral biofilms: untreated and *H. perforatum*-treated negative controls, 0.2% CHX-treated positive control, aPDT after rinsing off *H. perforatum*, and aPDT without rinsing off *H. perforatum*. The CFUs are presented on a log_10_ scale per square centimeter (log_10_/cm^2^). The p-values (t test) of the significantly different data are marked on the graphs.

Figure 4a shows the effects of aPDT using VIS + wIRA and *H. perforatum* on the log counts of the initial and mature oral biofilms after two hours and three days, respectively. The aPDT induced a complete eradication of the bacteria (p = 0.0001, Fig. 4a) within the initial biofilms treated with non-rinsed *H. perforatum*, whether followed or not by aPDT, as well as within the CHX-treated initial biofilms. Interestingly, the initial biofilms in which *H. perforatum* was rinsed prior to aPDT exhibited a mean of 0.69 ± 0.95 CFU in the log_10_ scale (median, 0.25), which is a significantly reduced (p = 0.002) microbial growth compared with the untreated negative control (mean, 3.81 ± 0.66; median, 3.75).

Figure 4(b,c) displays the microbial growth rates of the three-day-old oral biofilm after the application of aPDT. In the presence of *H. perforatum*, the aerobic and anaerobic microorganisms within the aPDT-treated biofilms showed no microbial growth and thus a CFU reduction of 100% (p = 0.0001). With regard to the aerobic biofilm bacteria, aPDT following rinsing of *H. perforatum* (mean, 5.49 ± 0.55; median, 5.52) yielded no significant CFU decline compared with the untreated negative control (mean, 6.23 ± 0.38; median, 6.17) and the CHX-treated positive control (mean, 5.44 ± 0.71; median, 5.45). Regarding the anaerobic biofilm bacteria, the application of aPDT after rinsing *H. perforatum* (mean, 4.49 ± 1.00; median, 4.64) failed to yield a decrease in the log_10_ CFU counts compared with the negative control (mean, 4.49 ± 1.06; median, 4.32), although it proved to be as effective as CHX (mean, 3.19 ± 2.17; median, 4.04). The application of *H. perforatum* only without subsequent aPDT had no significant effect on either aerobic (mean, 5.02 ± 1.18; median, 5.39) or anaerobic (mean, 5.33 ± 2.17; median, 4.04) microorganisms.

### Shift of bacterial spectrum after the use of aPDT against initial microbial adhesion

**Figure 5:**
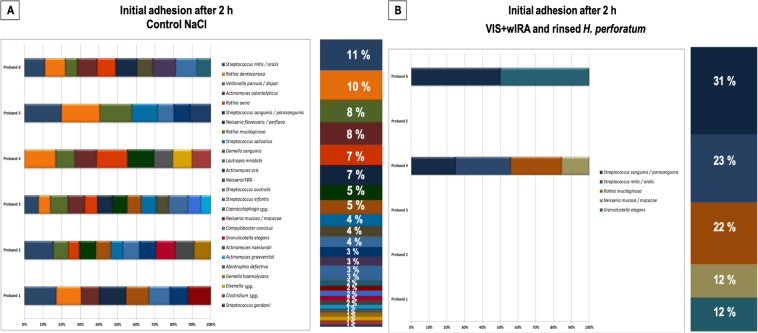
Relative distribution among the volunteers and total distribution (in percentages) of bacterial species among six probands after two-hour (2 h) initial adhesion following aPDT using VIS + wIRA and *H. perforatum*. Graph A depicts the bacterial composition of the untreated negative controls after initial adhesion. Graph B shows the bacterial diversity after aPDT against initially adhered bacteria. Consistent color coding was used for the study participants and bacterial species, as indicated by the schemes at the right of panels, respectively.

**Figure 6:**
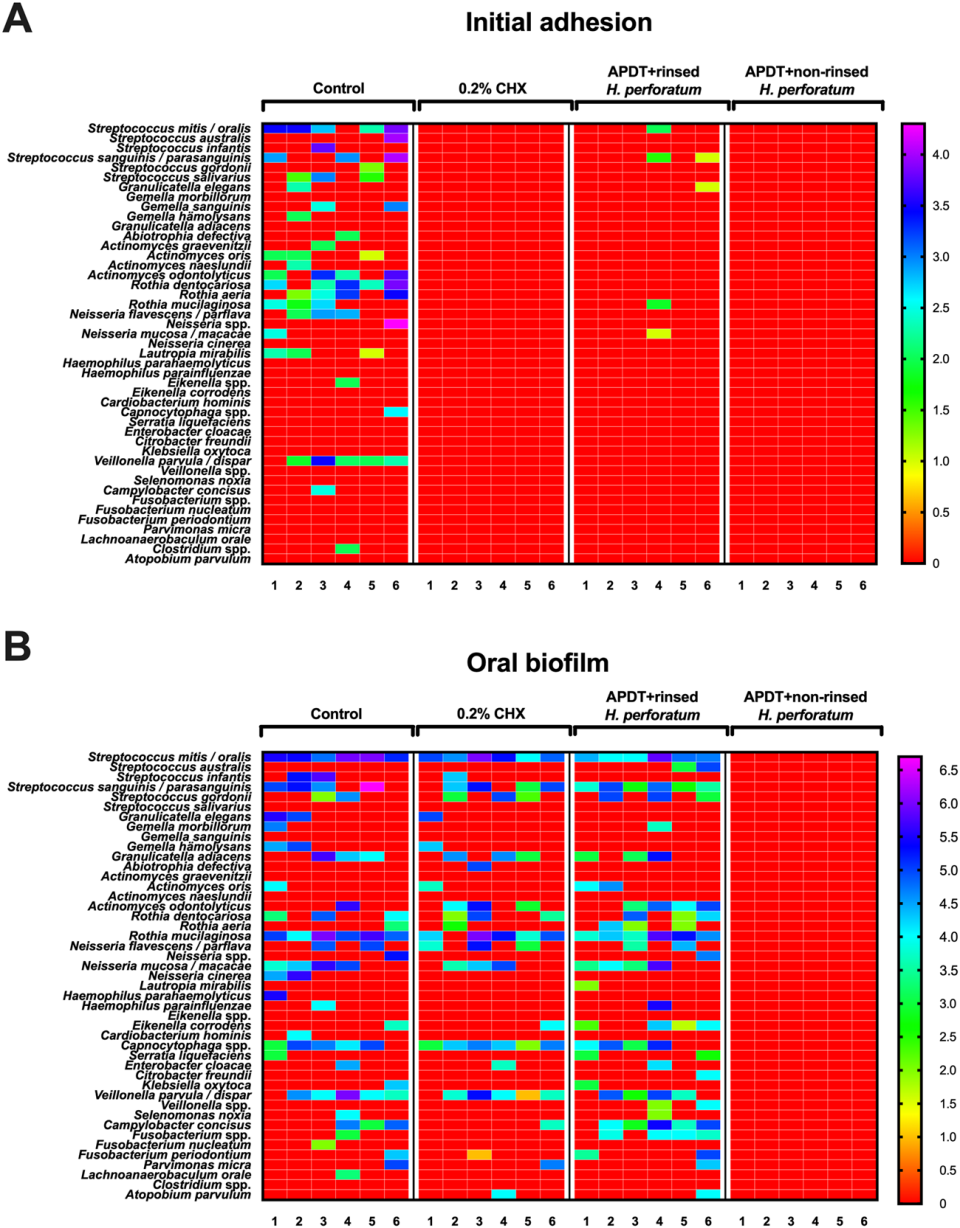
Heatmap presenting the absolute distribution (in log_10_/cm^2^) of bacterial species among six study probands after two-hour (2 h) initial adhesion and three-day (3 d) biofilm formation following aPDT using VIS + wIRA and *H. perforatum*. The photodynamic efficiency against oral microorganisms after initial adhesion and three-day biofilm formation is demonstrated in panels A and B, respectively. The bacterial composition of the following treatment groups is demonstrated for two-hour- and three-day old oral biofilms: untreated negative control, 0.2% CHX-treated positive control, aPDT after rinsing off *H. perforatum*, aPDT without rinsing off *H. perforatum*. Proband numbers for each treatment group (n = 6) are shown in columns and variables (bacterial species and genera) are shown in rows. The variations in the colors depicted on the color scale bars on the right indicate how data values change from very low to extremely high.

**Figure 7:**
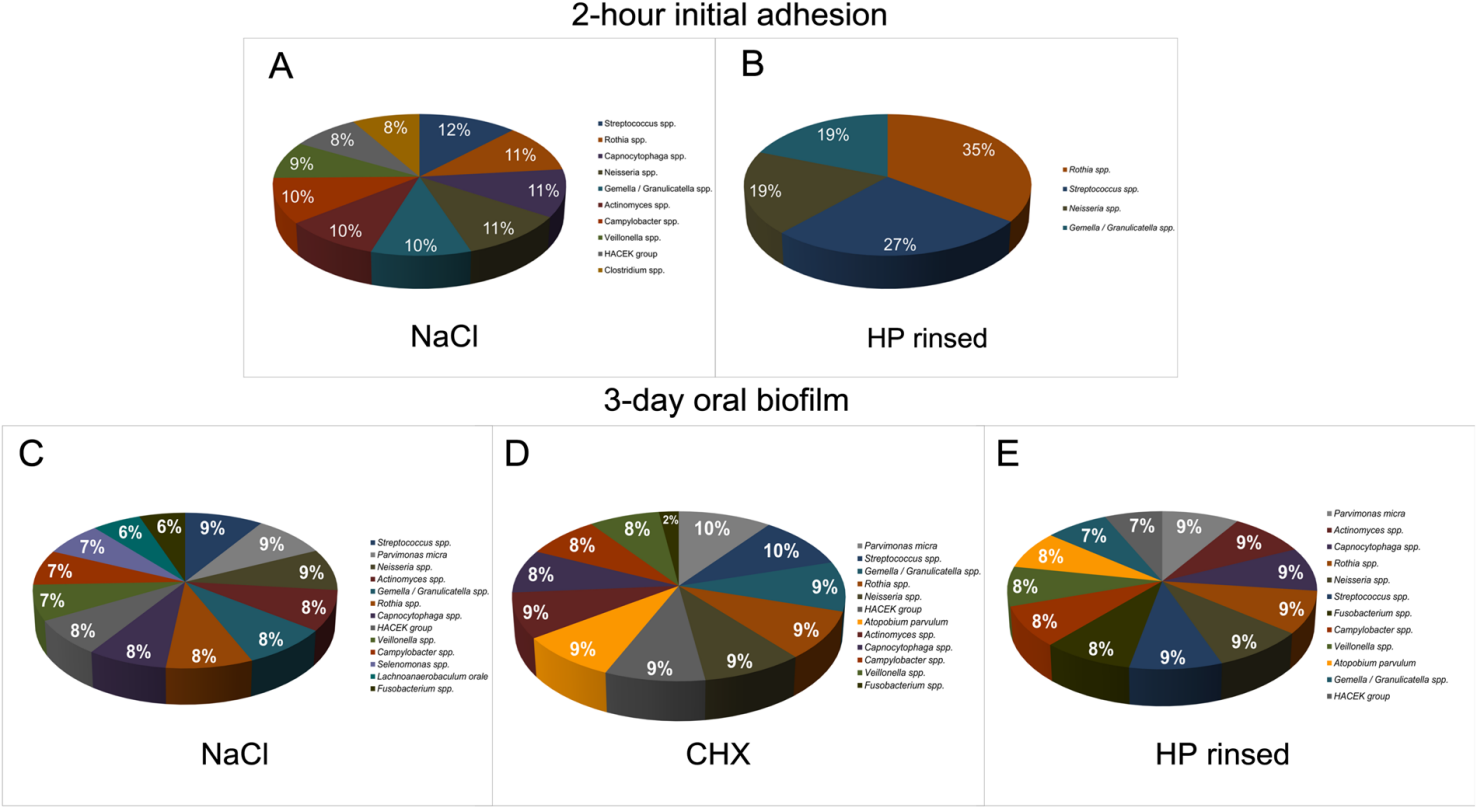
Pie charts demonstrating the total distribution (in percentages) of bacterial genera among six probands after two-hour (2 h) initial adhesion and three-day (3 d) biofilm formation following aPDT using VIS + wIRA and *H. perforatum*. Graph A depicts the bacterial composition of the untreated negative controls after initial adhesion. Graph B shows the bacterial diversity after aPDT against initial biofilms. Graphs C–E show the bacterial composition of three-day old biofilms divided into the following groups: the untreated negative control (C), after treatment with 0.2% CHX (D), and after aPDT (E) with VIS + wIRA and *H. perforatum* (was rinsed off prior to aPDT). Consistent color coding was used for the bacterial genera, as indicated by the schemes at the right of panels A–E, respectively.

The composition of cultivable bacteria in initial adhesion samples is analyzed in Figs. 5A,B, 6A, and 7A,B. After treatment with CHX (positive control) and *H. perforatum*, whether followed or not by aPDT, all bacteria were eliminated. From the original bacterial community in the untreated negative control, a total of 31 different cultivable species were isolated and identified, as shown in Fig. 5A. The microbial community was predominated (29%) by eight different streptococcal species (*Streptococcus mitis*/*oralis* 11%; *Streptococcus sanguinis*/*parasanguinis*, 7%; *Streptococcus salivarius*, 4%; *Streptococcus australis*, 3%; *Streptococcus infantis*, 3%; *Streptococcus gordonii*, 1%). Different species of the genera *Rothia (Rothia dentocariosa, Rothia aeria, Rothia mucilaginosa), Actinomyces* (*Actinomyces odontolyticus*, *Actinomyces graevenitzii, Actinomyces oris, Actinomyces naeslundii), Neisseria* (*Neisseria flavescens*/*perflava, Neisseria mucosa*, *Neisseria macacae)* and *Gemella* (*Gemella sanguinis, Gemella haemolysans)* were found in 22%, 14%, 10% and 5% of the isolated bacteria, respectively. The percentages of all other recovered species mainly belonging to the genera *Veillonella*, *Capnocytophaga* and *Campylobacter*, ranged from 2–8%. As depicted in Fig. 5B, the aPDT with VIS + wIRA and *H. perforatum –* which was rinsed prior to aPDT – reduced the number of viable bacterial species from 31 to 8, comprising *S. sanguinis*/*parasanguinis* (31%), *S. mitis*/*oralis* (23%), *R. mucilaginosa* (22%), *N. mucosa*/*macacae* (12%) and *Granulicatella elegans* (12%).

In the presence of reduced microbial richness, the species diversity of the surviving bacteria among the probands was also strongly affected. The most prevalent species in the untreated control group were *Streptococcus mitis*/*oralis, R. dentocariosa* and *Veillonella parvula*/*dispar* (detected in five volunteers), followed by *A. odontolyticus* and *R. aeria* (retrieved from four volunteers). All other species were detected in three volunteers or fewer. After pre-treatment with *H. perforatum*, rinsing and subsequent aPDT with VIS + wIRA, no cultivable bacteria were detected in four of the volunteers. The two remaining positive samples were mainly comprised of *S. sanguinis*/*parasanguinis* and *G. elegans*, whereas seven different species were only detectable in the second volunteer.

### Shift of bacterial community composition after aPDT application to the mature oral biofilm

**Figure 8:**
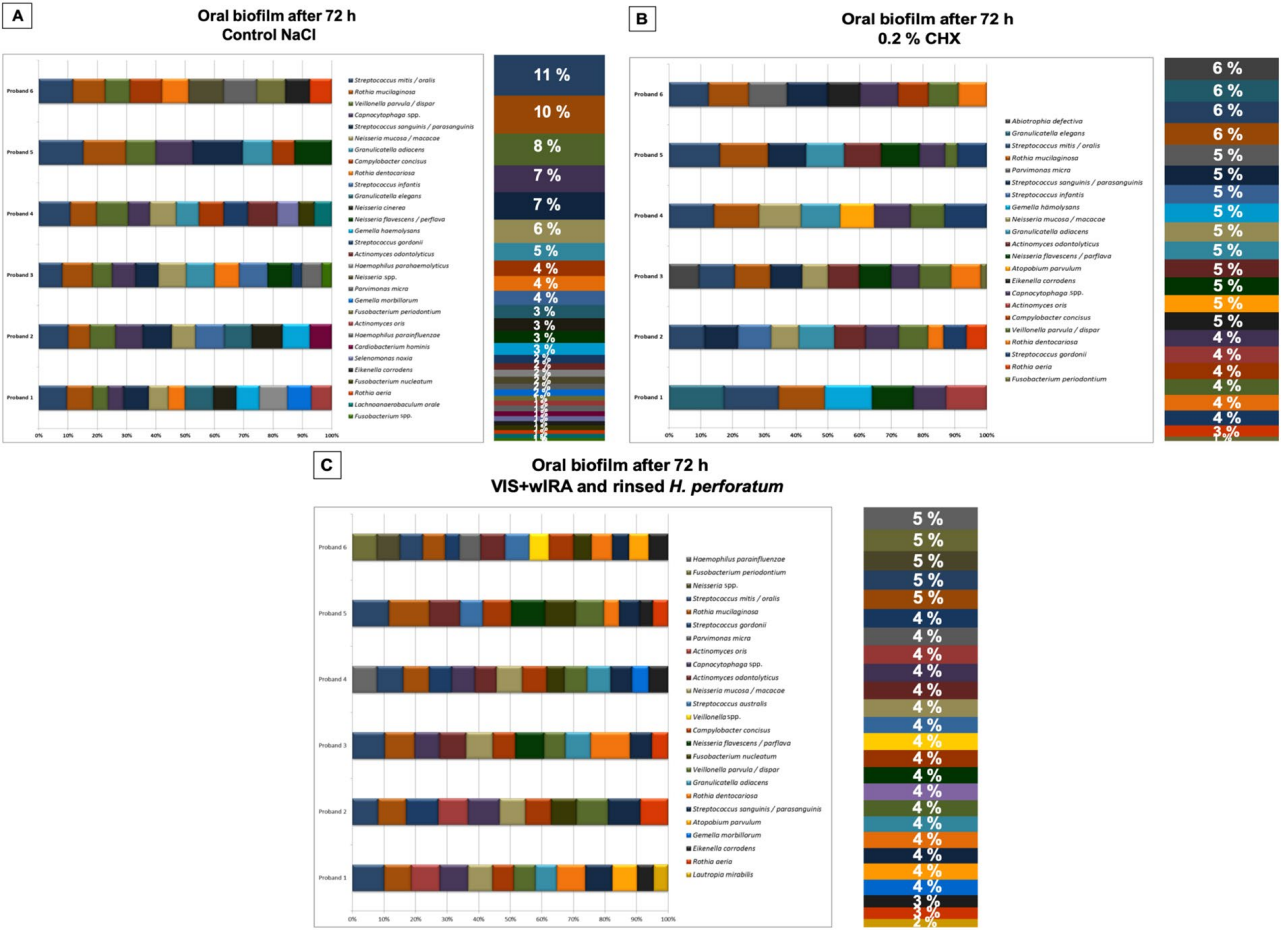
Relative distribution among the volunteers and total distribution (in percentages) of bacterial species among six probands after three-day (3 d) biofilm formation following aPDT using VIS + wIRA and *H. perforatum*. Graphs A–C demonstrate the bacterial composition of three-day old biofilms divided into the following groups: the untreated negative control (**A**), after treatment with 0.2% CHX (**B**), and after aPDT (**C**) with VIS + wIRA and *H. perforatum* (was rinsed off prior to aPDT). Consistent color coding was used for the study participants and bacterial species, as indicated by the schemes at the right of panels A–C, respectively.

Interestingly, after aPDT with VIS + wIRA in the presence of *H. perforatum*, the total microbial load of the biofilms was reduced by 100%. The microbial richness after pre-treatment with *H. perforatum* and aPDT with VIS + wIRA and the bacterial composition of the controls (negative: untreated, positive: treatment with 0.2% CHX) are illustrated in Figs. 8A–C, 6B and 7C–E. Regarding the microbial richness within the mature biofilms, the untreated control, the CHX-treated positive control and the aPDT-treated biofilms comprised 35, 27, and 30 different bacterial species, respectively.

The microbial community in the negative control group (Fig. 8A) was predominated (24%) by six different streptococcal species (*S. mitis*/*oralis*, 11%; *S. sanguinis*/*parasanguinis*, 7%; *S. infantis*, 4%; *S. gordonii*, 2%). Different species of the genera Rothia (*R. dentocariosa, R. aeria, R. mucilaginosa)*, Neisseria (*N. flavescens*/*perflava, N. mucosa*/*macacae, N. cinerea)*, *Veillonella* (*V. parvula*/*dispar*) and *Granulicatella* (*G. adiacens, G. elegans*) were found in 15%, 14%, 8% and 8% of the isolated bacteria, respectively. The percentages of all other recovered species mainly belonging to the genera *Capnocytophaga*, *Campylobacter*, *Gemella* (*G. sanguinis, G. haemolysans)* and *Actinomyces* (*A. odontolyticus*, *A. oris)* ranged from 3 to 7%.

In the positive control (Fig. 8B), the microbial richness was reduced to 27 species after treatment with 0.2% CHX. Although the counts of *Eikenella corrodens*, *S. gordonii*, and *R. dentocariosa* increased after the CHX treatment, all of the other bacterial counts within the positive control decreased. A total of ten species could not be detected after treatment with CHX, whereas *Abiotropha defectiva* and *Atopobium parvulum* could only be detected after the treatment with CHX.

As depicted in Fig. 8C, the aPDT with VIS + wIRA and *H. perforatum –* which was rinsed prior to aPDT – reduced the number of viable bacterial species from 35 to 30. The aPDT-treated biofilm mainly comprised 17% *Streptococcus* spp. (*S. mitis*/*oralis*, 5%; *S. sanguinis*/*parasanguinis*, 4%; *S. australis*, 4%; *S. gordonii*, 4%), 13% *Neisseria* spp. (*N. flavescens*/*perflava*, 4%; *N. mucosa*/*macacae*, 4%) and 12% *Rothia* spp. *(R. mucilaginosa*, 5%; *R. dentocariosa*, 4%; *R. aeria, 3*%). The percentages of the other detected genera such as *Haemophilus*, *Campylobacter*, *Gemella*, *Veillonella*, *Capnocytophaga* and *Actinomyces* varied from 4 to 5%. A total of 15 out of the 30 recovered bacterial species were found to decrease in number. Nine species – *Haemophilus parahaemolyticus, N. cinerea, Cardiobacterium hominis, Selenomonas noxia, Lachnoanaerobaculum orale*, *Fusobacterium* spp., *S. infantis, G. elegans* and *G. haemolysans* – completely disappeared after the aPDT treatment with *H. perforatum*, whereas *S. australis, Veillonella* spp., *Atopobium parvulum* and *Lautropia mirabilis* only appeared after the photodynamic application of *H. perforatum*.

Regarding the species distribution among the probands, *S. mitis*/*oralis*, *R. mucillaginosa* and *V. parvula*/*dispar* were detected in all six volunteers of the untreated control group, with a total distribution ranging from 8 to 11%. *Capnocytophaga* spp. (7%) was detected in five volunteers, whereas the other bacterial species were recovered from 1–4 probands. After the aPDT treatment, *S. mitis*/*oralis*, *S. sanguinis*/*parasanguinis, R. mucillaginosa, V. parvula*/dispar and *Campylobacter concisus* were detected in all six volunteers, with a total distribution ranging from 4 to 11%. All other species appeared in 1–5 volunteers and ranged from 1 to 4%. After aPDT treatment, a total of eight aerobic and three anaerobic microorganisms increased in their relative distribution compared with the CHX treatment, where only three aerobic microorganisms increased. In terms of the new appearance of bacteria after the aPDT with *H. perforatum*, two aerobic and two anaerobic bacteria emerged compared with the CHX treatment, where only one aerobic and one anaerobic bacteria appeared.

## Discussion

The present study has established an effective method for microbial photoinactivation combining VIS + wIRA with the natural photosensitizing agent *H. perforatum* to treat *in situ*-formed initial and mature oral biofilms for the first time. The aPDT combining VIS + wIRA and photosensitizing agents such as TB and Ce6 was reported to successfully photoinactivate oral pathogenic microorganisms situated even in the deepest niches of *in situ* supragingival and subgingival biofilms in earlier own reports^14,15,17,33^. In an *in vitro* study investigating the effectiveness of *H. hypericum*-mediated aPDT on *E. faecalis* culture in planktonic form and biofilm, a significant microbial reduction of up to 5 log counts was detected^34^. The innovative contribution of this study is that, to the best of our knowledge, *H. perforatum –* widely known for its anti-depressant properties – is here studied for the first time in combination with VIS + wIRA as a light source to treat *in situ* oral biofilms^35–38^.

For an effective aPDT light source, visible light (VIS) with an accessory orange filter within the range of 570–780 nm was combined with water-filtered infrared-A (wIRA) to accentuate the amount of infrared-A radiation within the range of 780–1400 nm^39^. Since PS excitation is only possible within the range of VIS, the use of wIRA alone would not have yielded the desired photodynamic outcomes^39^. The advantages of using VIS + wIRA contrary to VIS alone have already been outlined in the literature^9^. The emission of energy in the form of light results in electron transfer reactions between the substrate and the PS, with the latter entering an electronically excited state^40^. The leading antimicrobial effect of aPDT is the subsequent production in elevated rates of reactive oxygen species (ROS) such as superoxide ion (O_2_^−^), hydrogen peroxide (H_2_O_2_) and free hydroxyl radicals (^.^HO), all yielded via Type I mechanisms^41^. In a Type II reaction, single oxygen radicals (^1^O_2_) are yielded after the reaction of triplet state PS and oxygen (O_2_)^42^.

Due to the short lifetime of ROS (about 10–320 ns), their diffusion after using conventional light sources (laser and non-laser) is limited and thus the direct aPDT-induced damage remains localized to the PS-infiltrated area^43^. VIS + wIRA also allows for the release of the protective protein ferritin^44^ to prevent cell destruction. The high safety of VIS + wIRA radiators can be guaranteed for treatment times of up to 30 min^45^, although 5 minutes proved to be a potent average aPDT duration in our report with a focus on daily clinical use. To facilitate a better distribution of photosensitizer within the bacterial cells and to achieve clinically meaningful treatment times, the biofilms were pre-exposed to *H. perforatum* for 2 min in the dark. In a systematic review, Fumes *et al*. described pre-irradiation times for methylene blue (MB) between 1 and 15 minutes, with optimal outcomes between 5 and 15 min^46^. However, there is no published data on the pre-irradiation incubation of *H. perforatum*, which exhibits enhanced cell penetration when diluted in ethanol^47^. Finally, VIS + wIRA raises levels of physiological activity of the regulatory enzyme cytochrome c oxidase with absorption maxima at 620, 680, 760 and 825 nm^48^.

Due to its numerous advantageous properties, *H. perforatum* constitutes a multipurpose plant^49^ and is considered one of the most well-known natural photosensitizers in different medical fields^50^. Interestingly, *H. perforatum* has proven extremely effective in the treatment of non-melanoma skin cancer as well as other dermal and internal cancer species^18,51,52^. Besides its excellent anti-oxidant and anti-inflammatory effects^53^, *H. perforatum* has been successfully applied in dentistry for the pain management of trigeminal neuralgia^54^. Furthermore, hypericin and hyperforin – the main components of *H. perforatum* – are responsible for enhanced anti-bacterial activity against Gram-positive and Gram-negative microorganisms in both planktonic and biofilm forms^55^. As a result of its beneficial features, *H. perforatum* can be integrated in a greater number of clinical photoinactivation protocols compared with Ce6, TB, ICG and MB^18,35,37,56^. When administered systematically, *H. perforatum* inevitably interacts with several cytochrome enzymes, inducing lower plasma levels of anti-cancer agents or other medicines^57^. Nevertheless, local application of *H. perforatum* is associated with fewer side effects regarding cytotoxicity and cross-reactivity with other medicaments^58^. Even after the combined oral application of *H. perforatum* using both a single- and double-dose of UV radiation, visible light, and solar-simulated radiation, no significant pathogenic alterations could be detected at erythema threshold levels^59^. However, a recent report highlighted the increase of round fibroblastic cells, which indicated high cytotoxicity in concentrations of *H. perforatum* higher than 10–50 mg/ml^60^.

Solvent extraction is the most common means of extracting Hypericum from the flowers of *H. perforatum* (St John’s Wort). Solvents include methanol, ethanol, ethyl acetate and acetone. Scientific studies indicate when using extraction solvents containing more than 50% ethanol or methanol in water the content of the flavonoids in particular Hypericum and Hypericin in the extracts is similar independent of the extraction solvent^61^. *H. perforatum* extracts are often purified by removing the solvent and then dried into powders prior to commercial sale. This project required a crude liquid extract which contained the solvent for further clinical trials. For this reason methanol, ethyl acetate and acetone were not used as the solvent for the extraction of Hypericum due to the potential toxicity of the solvent in the crude extract. In order to extract bioactive constituents from *H. perforatum*, organic solvents were preferred, as ethanolic *H. perforatum* extracts showed the highest anti-bacterial potential against diverse Gram-positive and Gram-negative bacteria^37^. In the literature, other *H. perforatum* hydroethanolic extracts such as tinctures with a high alcohol content ranging from 45 to 50% have been widely used in complementary medicine^62^. In a recent report, 60% or above of ethanol was utilized to obtain the highest yields of lipophilic hyperforin^63^. However, the use of 100% (absolute) ethanol led to a reduction of specific hydrophilic components such as flavonoids^18^. Since bacteria in biofilms are more resistant to antimicrobials than their planktonic counterparts, we did not expect a significant decrease of biofilm viability after its exposure to 16% alcohol solution for seven minutes as detected by CFU. To confirm our assumption, we ran primary experiments resulting in no significant differences in CFUs between biofilms after 7-min treatment with 16% alcohol and the untreated control (Supplementary Fig. S1). Hence, a bactericidal effect of alcohol at the tested concentration can be excluded. It is recommended that future studies examine the role of alcohol in detail using live dead staining along with CFU determination. A bactericidal effect of the alcohol on the mature oral biofilm is not possible after an incubation time of seven minutes as detected by the CFU^64^. The authors reported that an alcohol concentration of 30% and incubation time of 8 hours were required to detect bactericidal effects against biofilms formed by *Klebsiella pneumoniae* and *Pseudomonas aeruginosa* after 40 hours. It should be taken into consideration that mature multispecies oral biofilms are more resistant to alcohol than mono-species biofilms tested in the aforementioned study. Ultrasonication (70% amplitude) using a digital ultrasonic bath (Sonorex Digital 10 P from Bandelin, Berlin, Germany) was applied to dissolve adherent bacteria from substratum. This ultrasonication power treatment for 4 min on ice was highly effective in killing adherent anaerobic bacteria such as *Porphyromonas gingivalis*, as reported in a previous own study^65^.

In a recent study, a promoting effect for periodontal healing was found for photosensitizer methylene blue (MB), which was dissolved in 20% ethanol during aPDT^13,47^. Presumably, this can be attributed to the up to 5-fold increase in survival time of ROS^66^. Another study showed that 20–30% ethanol is necessary to prevent the resuscitation of multispecies biofilms^67^. This supportive antimicrobial effect of ethanol can be attributed to the manipulation of the bacterial membrane and the denaturation of proteins^68^. In earlier reports, it has been shown that lipophilic photosensitizers are much more efficient than hydrophilic ones with the same ROS supply^69,70^. In contrast to conventional photosensitizing agents such as TB, ICG, MB and Ce6, the high anti-bacterial efficiency of *H. perforatum* extract cannot be attributed to mechanisms involving only one specific pure substance. Interestingly, synergetic interactions between three main constituents – namely hypericin, pseudohyperin and hyperforin – are responsible for the high antimicrobial potential of *H. perforatum*^26,27,71,72^. Nevertheless, their exact mechanism of action remains unknown^26,27^. Further studies are necessary to determine the synergistic potential of specific *H. perforatum* components.

Obtaining an *in situ* oral biofilm enabled us to conduct the assays under more realistic experimental conditions, as already described in previous own reports^73,74^. Bovine enamel slabs (BES) represent a favorable habitat for the adhesion of oral microorganisms which is physicochemically similar to human tooth surfaces^75^. Due to substantial differences in biofilm composition and architecture between artificial and natural tooth surfaces, the tests were conducted solely on the latter.

About 700 oral bacterial species are estimated as residing within the oral cavity, of which only 50% have been cultivated thus far^76,77^. Subsequently, the plate count technique as well as the culture media favor the growth of certain types of bacteria^78^. Nevertheless, CFU remains the most widely used gold standard method for the quantification of viable bacteria^79^, which is the focus of this study.

The application of aPDT completely inhibited bacterial growth when the initial and three-day-old biofilms were photodynamically treated in the presence of *H. perforatum*.

This is a very interesting finding, indicating the high bactericidal potential of *H. perforatum*-mediated aPDT compared with aPDT employing other photosensitizers in previous reports^17,76,77,80^. The presence of extracellular polymeric substances (EPS) within the oral biofilm and the chemical composition of the photosensitizers and cell envelope in Gram-positive and Gram-negative bacteria are crucial parameters that can define the degree of photosensitizer diffusion in biofilm biomass. In order to investigate the exact depth of penetration as well as whether non-viable bacteria can survive this procedure, further studies are necessary. Another critical aspect is the possibility that aPDT favors the survival of resistant bacteria. Although there are no relevant studies in the literature, even if this assumption was true, the application of low concentrations of photosensitizer would carry the same risk of creating a resistance-prone microbial community as treatment with low doses of CHX or antibiotics^1,2,81,82^.

In this study, we have shown that treatment with CHX as well as aPDT after rinsing off *H. perforatum* reduced the number of viable bacterial species, albeit not significantly. Nevertheless, the shift to a predominately aerobic environment after aPDT holds strong importance as it implies the presence of a high alteration potential of *H. perforatum*-mediated aPDT on biofilm synthesis. Caries and periodontitis are biofilm-associated oral infections that can be controlled after favorably changing the composition of the pathogenic microbial ecosystems into healthier microbial communities^23,77,83,84^. Ascertaining which alterations of the biofilm composition are advantageous for the development of a ‘healthy’ microbial environment constitutes an alternative treatment approach in case the complete mechanical removal of the biofilm poses clinical challenges and should be the focus of future investigations^85–87^.

Interestingly, *H. perforatum* had the highest efficiency as a photosensitizer when it was not rinsed prior to aPDT. This could be explained by the fact that the prolonged contact of *H. perforatum* with the oral biofilm could facilitate the maximum penetration of the biofilm through diffusion or endocytosis^4^. Taking the short lifetime of the ROS (about 10–320 ns) into consideration, the time extension of the synergistic interaction between diverse components in *H. perforatum* total extract and biofilm constituents such as EPS may be crucial for the aPDT-induced antimicrobial efficacy^17,21^. The highest antimicrobial effect can be achieved when the photosensitizer remains as close as possible to the targeted bacteria^43,88^.

To sum up, this study has shown that *H. perforatum* as a PS in combination with VIS and wIRA has a strong ability to eradicate *in situ* oral biofilms. The null hypothesis was rejected. The results of the study highlight the already-known positive effects of aPDT in the dental field and introduce *H. perforatum* as a potent photosensitizer for clinical use.

## Material and Methods

### Selection of study volunteers and test samples

After the review and approval of the study protocol by the Ethics Committee of the University of Freiburg (Nr. 91/13), six healthy volunteers between the age of 24 and 44 were selected and gave their written informed consent to participate in this study. All experiments and data collection were performed in accordance with relevant institutional and national guidelines and regulations. Individual clinical oral examinations were performed for each volunteer prior to study enrollment. Thus, the salivary flow rate was estimated in the range of 1.2 ± 0.4 ml/min, and the lactate production rate was measured at 2.7 ± 0.6 (scale from 1 to 9). The mean values for decayed, missing, filled teeth (DMFT) ranged between 0 and 7^17,89^. A complete dentition allowing for sufficient room for the slabs served as a requirement for the stable maintenance of the splint. The exclusion criteria included severe systematic disease, diseases of the salivary glands, carious lesions or periodontal disease, pregnancy or lactation, and use of antibiotics or local antimicrobial mouth rinses e.g. chlorhexidine (CHX) within the last 30 days^17,80^.

In order to perform the experiment, BES were obtained from freshly slaughtered two-year-old cattle (diameter, 5 mm; surface area, 19.63 mm²; height, 1 mm). Before extracting their teeth, the cattle were examined for the absence of bovine spo.pngorm encephalopathy (BSE) using an IDEXX labor index diagnostic kit (Ludwigsburg, Germany). The BES were fixed into plastic holders yielding parallel surfaces with an average height of 1 mm and were then polished using wet sandpaper with increasing corn sizes from 250 to 40,000 grits in a special polishing machine (Knuth-Rothor-3; Streuers, Willich, Germany) to yield a smooth surface. To guarantee consistency and thereby confirm the presence of intact enamel with clean polished surfaces a light microscope (Wild M3Z, Leica GmbH, Wetzlar, Germany) was used, and the BES were finally disinfected as previously described^80^. In brief, the first disinfection step was ultrasonication of the BES for 3 minutes with NaOCl (3%), followed by air drying to remove the superficial smear layer. For additional disinfection, 70% ethanol was subsequently applied for three minutes under ultrasonication. In the second disinfection step, BES were fixed to acrylic oral appliances described elsewhere and both BES and the devices were ultrasonicated twice in double-distilled water for ten minutes to allow the removal of residual NaOCl. Before the experiments, the BES-loaded oral devices were stored in distilled water for at least 24 hours to hydrate^17^.

Individual upper jaw acrylic devices were constructed with a total of six round openings at the interdental area between the upper premolars and molars, three on each side, respectively. The openings were sufficiently wide and deep to allow for the insertion of BES without interaction with the mucosa (Fig. 1). In order to fix the BES into the openings, an A silicon compound (Panasil initial contact X-Light, Kettenbach GmbH & Co. KG, Eschenburg, Germany) was used. In order to ensure that only BES surfaces were exposed to the oral cavity and that no unwanted contact took place between the buccal mucosa or the tongue and the BES, the BES margins were fully covered with silicon. In order to yield a sufficient number of biofilm samples for the aPDT assays, each volunteer carried the appliances loaded with a total of twelve BES twice, for two hours and three days, respectively.

### Radiation source and photosensitizer

The obtained biofilm samples were illuminated by broadband VIS + wIRA radiator (Hydrosun 750 FS, Hydrosun Medizintechnik GmbH, Müllheim, Germany) containing a 7 mm water cuvette^14,17^. For the absorption of the infrared-B and -C wavelengths emitted by the halogen lamp, an accessory water filter (dimensions: length: 28 cm, width: 27 cm, height: 28 cm) using 750 W (voltage: 230 V, 50–60 Hz) was inserted into the light path. The radiator also included an orange filter, namely BTE 31, with a diameter of 10 cm and an efficiency level of 200 mW/cm². The BTE 31 filter enabled more than a doubled-weighted efficient integral illumination with reference to the absorption spectrum of protoporphyrin IX. In order to prevent superficial overheating, the absorption bands 944 and 1180 nm were also filtered. The total spectrum of the water-filtered absorption ranged from 570 nm to 1400 nm, with local minima at around 970 nm, 1200 nm and 1430 nm, respectively^90^. The total amount of unweighted (absolute) radiation applied to the biofilms for 5 minutes equaled 200 mW cm^−2^ VIS + wIRA, which comprised 48 mW cm^−2^ VIS and 152 mW cm^−2^ wIRA. This broad-spectrum light source provided optimal absorption for the tested photosensitizer, namely *H. perforatum* ethanol (15.8%) extract (St. John’s Wort, Quality Herbal Extracts, Cooranbong, Australia). Pseudohypericin – the main component of *H. perforatum –* can be activated within the UVA (and UVA1) visible absorption range, while absorption peaks are mainly exhibited at red wavelengths (570–700 nm)^91^. The visible absorption maximum (λ _max_) of *H. perforatum* is 598 nm^92^. For the assays, *H. perforatum* extract was diluted in 0.9% saline (NaCl) to a final concentration of 32 mg ml^−1^ and a final alcohol concentration of 15.8% (v: v). Prior to use, the original extract solution was stored in a light safety glass at 4 °C in the dark for up to fourteen days to block any light-induced photochemical attenuation.

### Extraction of *H. perforatum*

Wild-growing *Hypericum perforatum* was harvested during the flowering phase. The herbaceous material was dried, cut, and stored until further processing. The crude extract was produced by percolation using 95% aqueous ethanol as a solvent (menstruum). The milled herbal material was moistened with some of the menstruum and packed firmly in a cylindrical percolator. Additional menstruum was added to the percolator and the herb was allowed to macerate for 24 hours before the percolation was commenced. Percolation comprised the slow passage of menstruum through the column of drug. The maceration and percolation were performed at 30 °C. The percolation was repeated until the material was exhausted. The extractions were combined and the solvent recovered under vacuum evaporation to yield a final ratio of one part drug per one part solvent (1: 1).

### Ultra-high-performance liquid chromatography coupled with high resolution mass spectrometry (UPLC-HRMS)

The *H. perforatum* extract was submitted to UPLC-HRMS/MS analysis on an AQUITY system (Waters) connected to an LTQ-OrbitrapR XL hybrid mass spectrometer (Thermo Scientific) equipped with an electrospray ionization (ESI) source and operated in negative mode. A UPLC separation gradient was developed to efficiently resolve all compounds for a qualitative analysis. The flow rate was set at 0.4 mL/min and the solvent system was (A) water 0.1% formic acid and (B) acetonitrile 0.1% formic acid. The elution program was 2% B for 2 minutes, 100% B for 18 minutes, and held for 2 minutes. After returning to 2% B for 1 minute, column equilibration was performed for 4 minutes at the end of the run. The injection volume was set to 10 μL and samples were injected at 0.2 mg/mL in water-acetonitrile solution (1:1) on a Fortis, C18 (100 × 2.1 mm i.d, 1.7 μm particle size). The HRMS and HRMS/MS data were acquired in negative mode over a 100–1000 m/z range. The MS profile was recorded in full-scan mode (scan time = 1 micro scans and maximum inject time = 500 ms). The ESI conditions were as follows: capillary temperature 320 °C; capillary voltage −40 V; tube lens −120 V; ESI voltage 2.7 kV. Nitrogen was used as sheath gas (40 Au) and auxiliary gas (8 Au). For the HRMS/MS acquisitions, a data-dependent method including the detection (full scan) and fragmentation of the three most intense peaks per scan was used. The mass resolving power was 30,000 for both levels and the normalized collision energy (CID) in the ion trap was set to 35.0% (q = 0.25) for the HRMS/MS experiments. Chromatographic and spectrometric features were used for identifying extracts constituents such as retention time (Rt), polarity, accurate m/z, proposed elemental composition (EC) and ring double bond equivalent (RDBeq) values as well as HRMS/MS spectra and derived fragmentation motifs. The raw data were acquired and processed using XCalibur 2.2.4 software from Thermo Scientific.

### aPDT protocol for *in situ* oral biofilms

Each participant twice carried an individual BES-loaded upper jaw acrylic device for the assays involving the initial adhesion (two hours) and biofilm formation (three days). After obtaining the *in situ* biofilms, the devices were removed from the oral cavity. Subsequently, BES were detached using sterile tweezers and a dental probe to avoid disturbing the intact *in situ* biofilms. Each of the BES was rinsed for 30 seconds with sterile 0.9% NaCl to ensure the displacement of non-adherent bacteria. One BES served as a positive control and was treated with 0.2% CHX, while another two BES treated with 0.9% NaCl and *H. perforatum* without aPDT were used as negative controls. One biofilm-covered BES was preincubated in *H. perforatum*, which was rinsed off for 30 seconds with 1 ml 0.9% NaCl prior to aPDT *ex vivo* using VIS + wIRA to allow for optimal depth of the irradiation. The remaining BES was also preincubated in *H. perforatum*, which remained during aPDT *ex vivo* using VIS + wIRA. The preincubation time for each BES in *H. perforatum* at a concentration of 32 mg ml^−1^ was two minutes in multiwell plates (24-well plate, Greiner bio-one GmbH, Frickenhausen, Germany) in the dark as described elsewhere^17^, while the irradiation by VIS + wIRA lasted five minutes at 37 °C (Fig. 2). After aPDT, the BES were inserted into multiwell plates with 1 ml 0.9% NaCl and the quantification and identification of adherent microorganisms were performed by the CFU and with MALDI-TOF, respectively. The activity of the photosensitizer remained stable for six months of storage at low temperature (3 °C) in the dark using a light-tight bottle. The participants carried the BES twice for 72 h and 2 h, respectively.

### Quantification of the adherent oral microorganisms within biofilms

The margins and the bottom dentine surfaces of the biofilm-loaded BES were brushed down with small sterile foam pellets (Voco GmbH, Cuxhaven, Germany) to displace non-adherent microorganisms. Subsequently, the BES were washed with 0.9% NaCl for at least 10 seconds to remove the remaining non-attached microorganisms. For the dilution series, each treated BES was transferred into Eppendorf tubes (Eppendorf GmbH, Wesseling-Berzdorf, Germany) containing 1 ml 0.9% NaCl, ultrasonicated for 3 minutes in 1 ml NaCl on ice and finally vortexed for at least 30 seconds to dislodge the biofilm microorganisms from the BES surface. Subsequently, the suspensions untreated BES (negative control), CHX-treated BES (positive control) and all aPDT-treated BES were diluted from up to 1:10^3^ (initial biofilm) and up to 1:10^6^ (mature biofilm) in 0.9% NaCl. From each dilution series of the differently treated BES three different dilutions were plated on separate agar plates. Thereafter, the microorganisms were cultivated and identified as previously described^93^. In brief, for the CFU estimation of the aerobic and facultative anaerobic bacteria Columbia blood agar plates (CBA; Becton, Dickinson, Heidelberg, Germany) were used at 37 °C and 5% CO_2_ for 3–5 days. Anaerobic bacteria were cultivated on yeast-cystein blood agar plates (HCB; Becton, Dickinson, Heidelberg, Germany) and incubated for ten days at 37 °C (anaerobic chamber; Genbox bioMérieux SA, Marcy l’Etoile, France). The CFU were finally counted per ml of the original suspension using Gel Doc EQ universal hood (Bio-Rad Life Science Group, Hercules, CA, USA). Each measurement was repeated, and the surviving bacteria were counted by eye if necessary.

### Identification of the adherent oral microorganisms within biofilms

The recovered bacterial colonies were sub-cultivated to yield pure cultures. In order to identify certain bacterial species, MALDI-TOF analysis in a MALDI Biotyper Microflex LT (Maldi Biotyper, Bruker Daltonik GmbH, Bremen, Germany) was applied. The Biotyper 3.0 Software obtained mass spectra according to the manufacturer’s instructions and compared the acquired spectra with a total of 3,740 spectra of the reference database (representing 319 genera and 1,946 species). The resulting similarities were illustrated by a log score indicating the species level with values ≥ 2.0, whereas values ≥ 1.7 represented identification at the genus level. For values < 1.7, the yielded spectrum exhibited no significant similarity to any database spectrum. The analysis was repeated if the identification of the species could not be confirmed. Moreover, universal bacterial PCR was performed as a supplementary method to identify the surviving oral microorganisms. In the presence of MALDI-TOF scores < 1.7, the following primers were applied: TP16U1: 5′-AGAGTTTGATCMTGGCTCAG-3′ and RT16U6: 5′-ATTGTAGCACGTGTGTNCCCC-3′ followed by sequencing on a 3130 Genetic Analyzer (Applied Biosystems, Life Technologies GmbH, Darmstadt, Germany).

### Statistical analysis

The effects of aPDT using VIS + wIRA and *H. perforatum* as a photosensitizer were calculated in a detailed evaluation for all six volunteers. For descriptive analysis, mean and standard deviations were computed. For graphical demonstration of the results, boxplots were used. Diagrams that demonstrated the log_10_ scale per square centimeter (log_10_/cm^2^) were selected to visualize the viable bacteria stratified to biofilm age (initial/mature) and type of microorganism (aerobic/anaerobic). A Friedman test was used to depict the differences between the aPDT-treated groups and the controls. Because a nonparametric test lacks sufficient power due to the small number of cases, paired t-tests with Bonferroni correction (multiple testing) were used for pairwise tests of differences among biofilms. Scheffé’s method was applied to correct the multiple testing errors through the adjustment of p-values. The statistical analysis was performed using STATA 14.

## Supplementary information

Supplementary Fig. 1.

## Data Availability

All data generated or analyzed during this study are included in this published article.
