# Supplementary material for: Antimicrobial Photoinactivation Using Visible Light Plus Water-Filtered Infrared-A (VIS + wIRA) and Hypericum Perforatum Modifies In Situ Oral Biofilms
Source: Sci Rep. 2019 Dec 30;9:20325. doi: 10.1038/s41598-019-56925-7 (PMC6937260; doi:10.1038/s41598-019-56925-7)
Supplement: Supplementary file 1 — Supplementary Fig. 1. [file 41598_2019_56925_MOESM1_ESM.pdf]

Antimicrobial Photoinactivation Using Visible Light Plus Water-Filtered Infrared-A (VIS + wIRA) and *Hypericum Perforatum* Modifies *In Situ* Oral Biofilms

Andreas Vollmer[1], Ali Al-Ahmad[1], Aikaterini Argyropoulou[2], Thomas Thurnheer[3], Elmar Hellwig[1], Thomas Attin[3], Kirstin Vach[4], Annette Wittmer[5], Kerry Ferguson[6], Alexios Leandros Skaltsounis[2], Lamprini Karygianni[3*]

[1]Department of Operative Dentistry and Periodontology, Center for Dental Medicine, Albert-Ludwigs-University, Freiburg, Germany

[2]Department of Pharmacognosy and Chemistry of Natural Products, Faculty of Pharmacy, National and Kapodistrian University of Athens, Athens, Greece

[3]Clinic for Conservative and Preventive Dentistry, Center of Dental Medicine, University of Zurich, Switzerland

[4]Institute for Medical Biometry and Statistics, Center for Medical Biometry and Medical Informatics, Albert-Ludwigs-University, Freiburg, Germany

[5]Institute of Medical Microbiology and Hygiene, Albert-Ludwigs-University, Freiburg, Germany

[6]Botanical Innovation, Unit 2, 390 Clergate Road, Orange, NSW 2800, Australia

Running Title: Antimicrobial Photoinactivation of Oral Biofilms

***Corresponding author:**

Lamprini Karygianni

Clinic for Conservative and Preventive Dentistry, Center of Dental Medicine, University of Zurich, Switzerland

Phone Number: 0041 44 634 3275

E-mail: lamprini.karygianni@zzm.uzh.ch

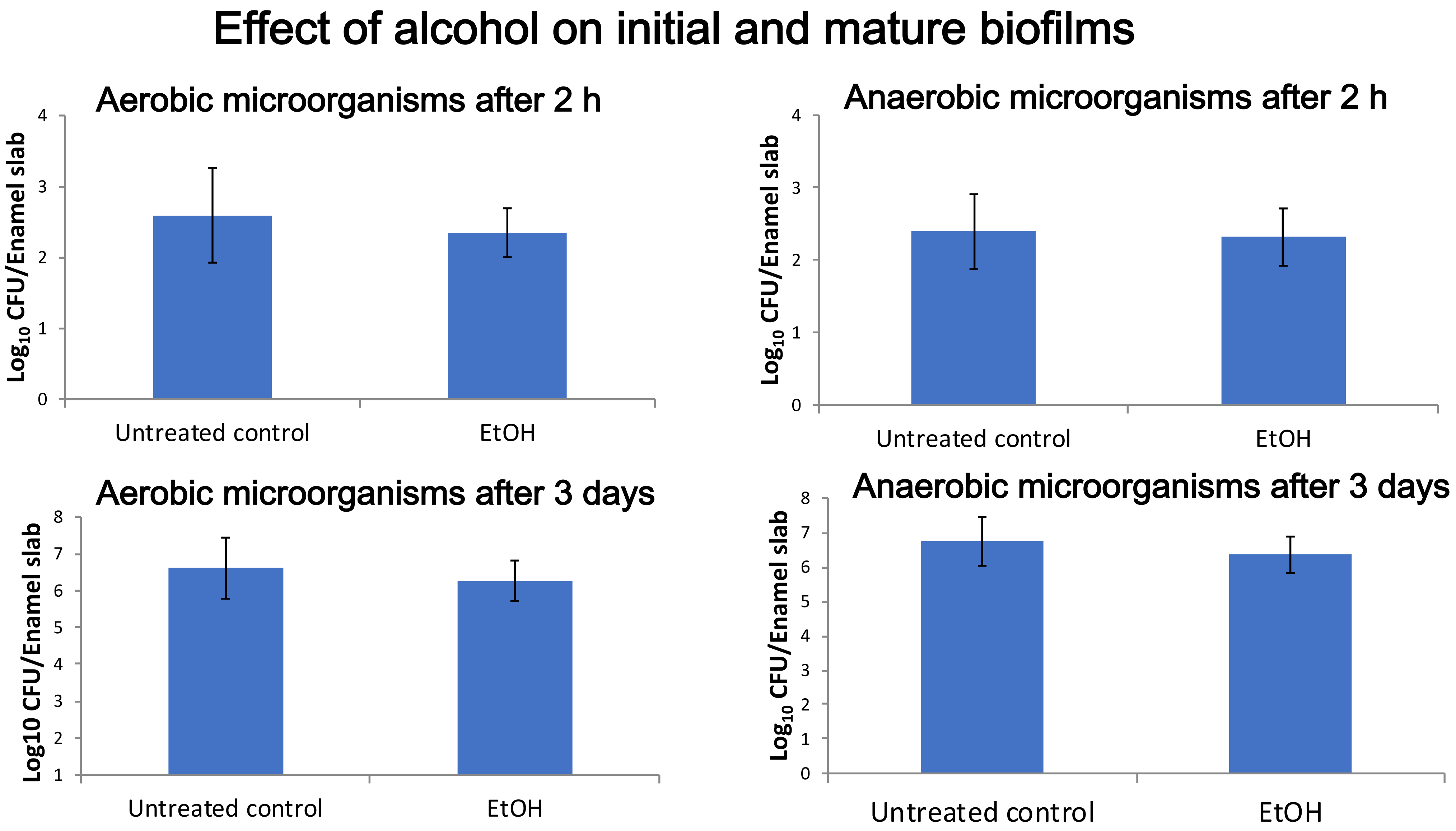

Supplementary Figure 1: Bar charts demonstrating the CFU values for initial (2h) and mature (3 days) biofilms treated with 16% (v:v) alcohol solution. Biofilms treated with 0.9% NaCl solution served as negative controls. Stardard deviations (SD) are marked on the graphs.
